# Ex vivo limb perfusion for traumatic amputation in military medicine

**DOI:** 10.1186/s40779-020-00250-y

**Published:** 2020-04-26

**Authors:** Alexander Kaltenborn, Nicco Krezdorn, Sebastian Hoffmann, André Gutcke, Kirsten Haastert-Talini, Peter M. Vogt, Axel Haverich, Bettina Wiegmann

**Affiliations:** 1 Department of Trauma and Orthopedic Surgery, Plastic, Hand and Reconstructive Surgery, Armed Forces Hospital Westerstede, Westerstede, Germany; 2 Department of Plastic, Aesthetic, Hand and Reconstructive Surgery, Hannover Medical School, Hannover, Germany; 3 Institute of Neuroanatomy and Cell Biology, Hannover Medical School, Hannover and Center for Systems Neuroscience (ZSN), Westerstede, Germany; 4 Department of Cardiothoracic, Transplantation and Vascular Surgery, Hannover Medical School, Hannover, Germany

## Abstract

**Background:**

Limb loss has a drastic impact on a patient’s life. Severe trauma to the extremities is common in current military conflicts. Among other aspects, “life before limb” damage control surgery hinders immediate replantation within the short post-traumatic timeframe, which is limited in part by the ischemic time for successful replantation. Ex vivo limb perfusion is currently being researched in animal models and shows promising results for its application in human limb replantation and allotransplantation.

**Presentation of the hypothesis:**

The current lack of replantation possibilities in military operations with high rates of amputation can be addressed with the development of a portable ex vivo limb perfusion device, as there are several opportunities present with the introduction of this technique on the horizon. We hypothesize that ex vivo limb perfusion will enable overcoming the critical ischemic time, provide surgical opportunities such as preparation of the stump and limb, allow for spare-part surgery, enable rigorous antibiotic treatment of the limb, reduce ischemia-reperfusion injuries, enable a tissue function assessment before replantation, and enable the development of large limb transplant programs.

**Testing the hypothesis:**

Data from in vivo studies in porcine models are limited by the relatively short perfusion time of 24 h. In the military setting, notably longer perfusion times need to be realized. Therefore, future animal studies must focus especially on long-term perfusion, since this represents the military setting, considering the time for stabilization of the patient until evacuation to a tertiary treatment center.

**Implications of the hypothesis:**

The development and clinical introduction of ex vivo limb perfusion in the military setting could lead to a drastic reduction in the number of limb amputations among service members. Ex vivo limb perfusion enables replantation surgery in Role 4 facilities and changes the clinical setting from a highly urgent, life-threatening situation to a highly methodical, well-prepared starting point for optimal treatment of the wounded service member. With its introduction, the principle of “life before limb” will change to “life before limb before elective replantation/allotransplantation after ex vivo limb perfusion”.

## Background

As early as 2008, a study from the United States predicted that by 2050, the number of amputations will increase by 72% [1]. The impact on the patents’ life and the socio-economic consequences of limb loss are remarkable. Patients eventually lose their jobs, and financial problems and social descent have been shown to be associated with limb loss, which often lead to psychological disorders (e.g., depression) [2].

The results of replantation surgery vary depending on the cause of the amputation, the patient’s condition and the quality of the specialized service provided. Furthermore, recent evidence underlines the role of allotransplantation of limbs in opposition to prosthetic limb replacement regarding functional outcomes and patient satisfaction [3, 4]. Replantation surgery is a highly elaborate microsurgical procedure that requires optimal interdisciplinary cooperation within an experienced team (for the aftercare as well). Therefore, the first line of treatment is limb salvage, though this is not feasible in every case. The technically demanding surgical realization of replantation is often not the only limiting factor, narrow time frames due to ischemia also need to be kept in mind [5]. The incidence of polytraumatized patients with life-threatening extremity injuries is increasing [5]. Especially in these cases, the strategy of “life before limb” damage control surgery within the short post-traumatic timeframe hinders replantation, which is partly defined by the limited ischemic time for successful replantation [4, 5]. This approach is basically equivalent between civilian and military trauma care.

There have been two major studies investigating the outcomes of reconstruction and amputation. The first was the Lower Extremity Assessment Project (LEAP) [6], which focused on civilian healthcare. The results suggest that the functional outcomes of reconstruction and amputation are at least similar among civilians being treated for major lower-extremity trauma [3, 4, 7]. Regardless of the type of treatment, LEAP outcomes were not optimal, with one-half of injured civilians reporting high levels of disability. Military-specific data were analyzed by the Major Extremity Trauma Research Consortium in their Military Extremity Trauma Amputation/Limb Salvage (METALS) study [8]. The METALS study focuses on major limb trauma in recent U.S. armed conflicts. In summary, most of the analyzed parameters mirror the LEAP results. Nevertheless, there are major differences between the clinical setting for civilian and military trauma amputation. The necessities of tactical evacuation in combat lead to longer ischemic times. Furthermore, the amputated limb is often highly contaminated with debris and dirt. Blast injuries especially are concomitant with a high degree of soft tissue trauma and a significant loss of tissue. Data from registries and recent studies indicate that approximately half of combat wounds inflicted in modern warfare are extremity injuries [9–11]. For severe injuries, either amputation or limb salvage procedures are the only options in the combat surgical setting, since replantation surgery is far too sophisticated and time-consuming under these conditions [12].

**Fig. 1:**
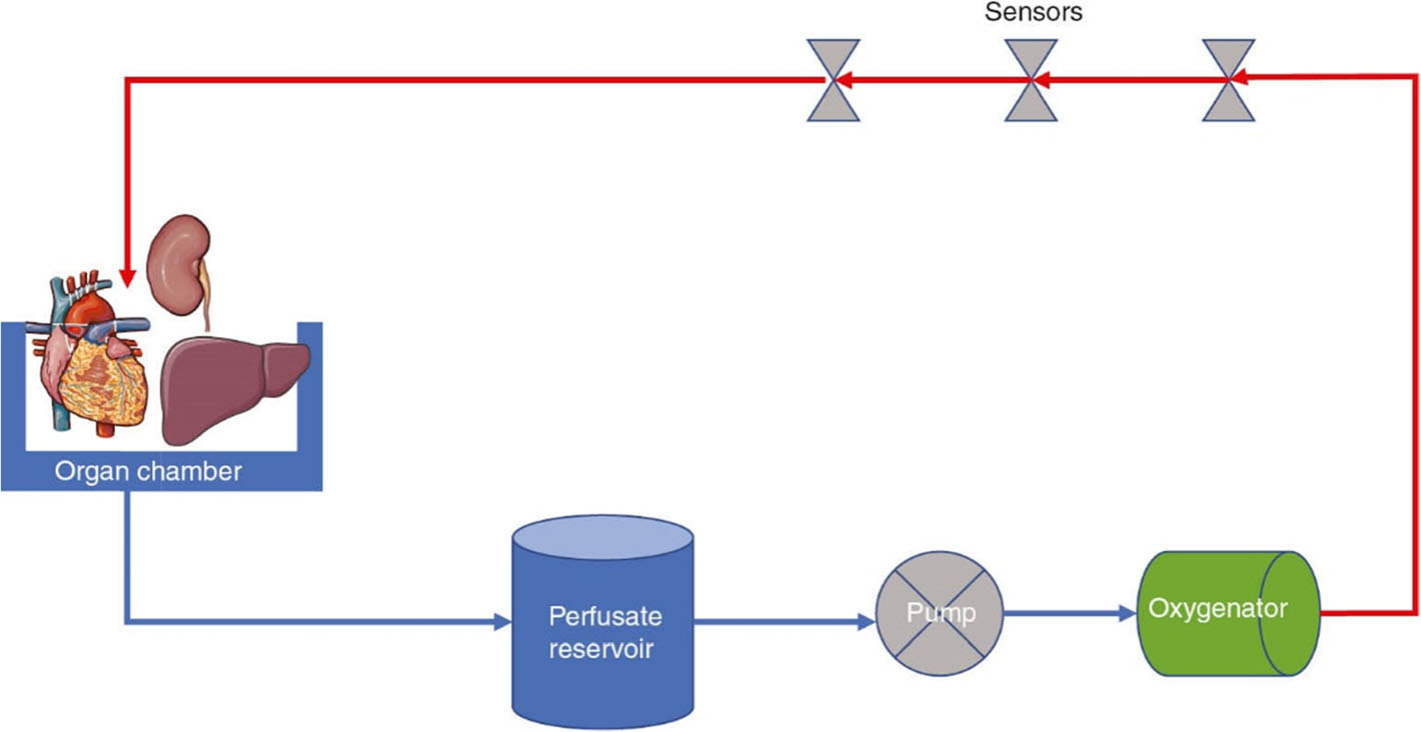
Basic schematic of a machine perfusion system*. *Figure by Krezdorn N, Tasigiorgos S, Wo L, Turk M, Lopdruo R, Kiwanuka H, et al. Tissue conservation for transplantation. Innov Surg Sci. 2017;2:171–87. 10.1515/iss-2017-0010 and used under the terms of the Creative Commons Attribution License

Our hypothetical treatment approach for this obvious military medical challenge is outlined in this paper. Recently, there has been growing evidence for the successful application of ex vivo perfusion in solid organ transplantation [13–17]. Figure 1 shows the basic scheme of a machine perfusion system. For example, ex vivo heart and lung perfusion can be applied for up to 8 and 12 h, respectively, in the clinical setting, and result in significantly better outcomes after transplantation compared to the gold standard of static cold organ preservation [14, 15]. Furthermore, the first large randomized clinical trial in liver transplantation was recently published and supports the aforementioned benefits of ex vivo organ perfusion [16]. In addition to the reduction of the cold ischemic time, ex vivo organ perfusion systems can be used for extended criteria donor organs due to an improvement of graft function during machine perfusion. Individualized organ therapy is possible following the reevaluation of the organ [17]. It can be assumed that ex vivo organ perfusion is able to relieve the donor organ shortage.

Ex vivo limb perfusion (EVLP) has also gained interest in recent years, although the evidence on EVLP is still scarce. In 2011, Constantinescu et al. showed the feasibility of a porcine model by perfusing swine limbs for up to 12 h with autologous blood [18]. The same group later allotransplanted a small series after 12 h of ex vivo perfusion and showed promising results regarding reperfusion injury and muscle contractility. Moreover, it could be shown that muscle contractility returned to nearly normal [19]. A Boston group analyzed seven forelimbs of pigs and divided these into two cohorts of cold perfusion compared to cold storage for 12 h [20]. In 2016, Ozer and colleagues subsequently increased the perfusion period to 24 h in a porcine model of eight limbs and applied normothermic perfusion with autologous blood [21]. However, a recent review of the current literature on this topic concluded that there is no standard protocol for EVLP, and it cannot be clearly stated whether the used perfusion solutions are optimal for experimental or clinical vascularized composite tissue allotransplantation (VCA) [22]. Additionally, in 2017, Werner and colleagues from the University of Michigan reported the ex situ perfusion of five human limb allografts from deceased heart-beating donors, which was feasible for up to 24 h with only small signs of tissue deterioration [23]. In summary, the limitations of the current literature are that there is no standardized perfusion protocol, and there are no data available indicating the effects of different perfusion solutions and temperatures, the various involved cell types (e.g., endothelial cells, nerve cells), or graft viability and functionality. Furthermore, a common problem in the aforementioned studies was weight gain during perfusion due to edema, but there are currently no data available about the impact of weight gain on subsequent replantation or transplantation, especially in humans. A selection of studies on ex vivo tissue perfusion is summarized in Table 1. Of note, all studies had perfusion times of no more than 24 h. In the military setting, the necessity for longer perfusion periods is highly likely due to strategic evacuations to Role 4 facilities. In our experience from the Afghanistan missions, the average time between damage control surgery in the Role 3 facility and strategic evacuation to the Role 4 facility in Germany was 48–72 h.

**Table 1:** Selection of previous studies on ex vivo perfusion in solid organs and extremities

Working group	Year of publication	Characteristics
Warnecke et al. (Hannover, Germany) [14]	2012	Pilot study on the human application of normothermic EVP in lung transplantation
Warnecke et al. (Hannover, Germany) [15]	2018	Results of the first randomized trial on the human application of normothermic EVP in lung transplantation
Nasralla et al. (United Kingdom) [16]	2018	Results of the first randomized trial on normothermic EVLP in liver transplantation
Constantinescu et al. (Bern, Switzerland) [18]	2011	Feasibility study of EVLP in a porcine model, 16 porcine forelimbs perfused for up to 12 h
Müller et al. (Bern, Switzerland) [19]	2013	EVLP in a porcine model, 64 forelimbs perfused for up to 12 h, replantation surgery was feasible without an increased risk for IRI
Kueckelhaus et al. (Boston, USA) [20]	2017	Developed a prototype hypothermic EVLP device, perfusion for 12 h with consecutive replantation in a porcine model
Ozer et al. (Ann Arbor, USA) [21]	2016	Porcine model with 20 pigs and EVLP up to 24 h, feasibility of extended limb survival with normothermic EVLP
Werner et al. (Ann Arbor, USA) [23]	2017	Near-normothermic EVLP in five human arms from organ donors for 24 h with shown tissue viability
Krezdorn et al. (Boston, USA; Hannover, Germany) [24]	2018	Normothermic EVLP in 8 porcine forelimbs for up to 12 h with subsequent replantation, notably lower expression of hypoxia-related genes in EVLP cohort

*EVP* ex vivo perfusion, *EVLP* ex vivo limb perfusion, *IRI* ischemia-reperfusion injury

### Presentation of the hypothesis

The current lack of replantation possibilities in military operations with high rates of amputation could be addressed with the development of a portable, clinically applicable EVLP device. There are several opportunities present with the introduction of EVLP on the horizon.

#### Critical ischemic time

First, EVLP is able to notably prolong the ischemic periods. After recovering the amputated limb, it can be conserved in the perfusion device under physiological conditions until the damage control surgery has been performed and the patient stabilized. Currently, the “life before limb” approach is unquestionably feasible and even recommended. Therefore, EVLP is associated with higher tactical and strategic flexibility. Strategic aeromedical evacuation can be planned and executed cautiously without the pressure of time, and definitive care in a Role 4 facility can be carefully planned.

#### Surgical opportunities

Before replantation, there is enough time for rigorous debridement of both the stump and the amputated limb to facilitate the successful re-adaptation of all the important anatomical structures. Further EVLP would allow for spare-part surgery of the perfused limb, even in cases of severe limb damage. As an example, in select cases, functional ankle joints could be used for knee joint replacement (van Nees procedure), or atypical osteomyocutaneous free flaps could be harvested from mangled perfused extremities.

#### Microbiological considerations

Combat-related injuries have been shown to be at great risk for contamination, especially with multi-resistant pathogens [25]. The reasons for the high incidence of infections with multi-resistant bacteria in combat wounds have yet to be satisfyingly identified [25]. Moreover, soil organisms and the amount of exposure to dirt during injury and tactical evacuation characterize the combat-related wound. EVLP enables the administration of high doses of antibiotic and antifungal medications without the constraints of systemic application [17]. Thus, these doses can be much higher than the regularly administered doses and can significantly decrease the risk of post-operative infections.

#### Ischemia and reperfusion-related injuries

Ischemia and reperfusion-related injuries (IRI) threaten not only the replanted limb, but due to a sterile systemic inflammation, also the patient’s life [26]. Ischemia leads to metabolic changes in the affected tissue, depending on the ischemic period, basically due to the shift from aerobic to anaerobic energy production. With reperfusion of the ischemic tissue, an immunological reaction concomitant with cell apoptosis and necrosis is observed. It has already been shown that the expression of hypoxia-related genes is less activated during ex vivo perfusion compared to static cold storage [24]. Thus, the reduction of IRI is a logical consequence of EVLP. Furthermore, anti-inflammatory and immunomodulatory measures can be applied during ex vivo perfusion. A good example for this pharmacological approach is the addition of prolyl hydroxylase domain (PHD) inhibitors, which have recently been shown to attenuate IRI in experimental models [27].

#### Assessment of tissue function.

Another great opportunity is the possibility to assess tissue function and vitality during EVLP. From experiences with normothermic machine perfusion for subsequent transplantation of the liver, it is already known that repeated evaluation of graft function and metabolism leads to higher rate of successful transplantation procedures [17]. It is possible that the tissue of amputated limbs could be reconditioned during EVLP, for example by the application of optimal oxygen levels and supplementation of nutrition.

#### Transplantation and Immunoconditioning

VCA is a further treatment option for cases in which replantation is not feasible (e.g., due to severe loss of tissue or a mangled extremity). However, the widespread application of VCA has not yet been realized. It requires life-long immunosuppression, which is associated with repercussions such as an increased risk of malignancy occurrence or renal failure [28, 29]. Recent data show promising results regarding the knockdown of major histocompatibility complex (MHC) molecules [30, 31]. This approach might be able to reduce the amount of post-transplant immunosuppression, as well as the early allograft rejection risk. Furthermore, a study that applied ex vivo perfusion in a VCA rat model showed a significant reduction of MHC I expression by at least 50% in all tissue compartments [30]. It was also found that knockdown prolonged rejection-free survival by 60% [30]. Comparable results have recently been reported in solid organ transplantation research [31].

#### Hypothetical application

**Fig. 2:**
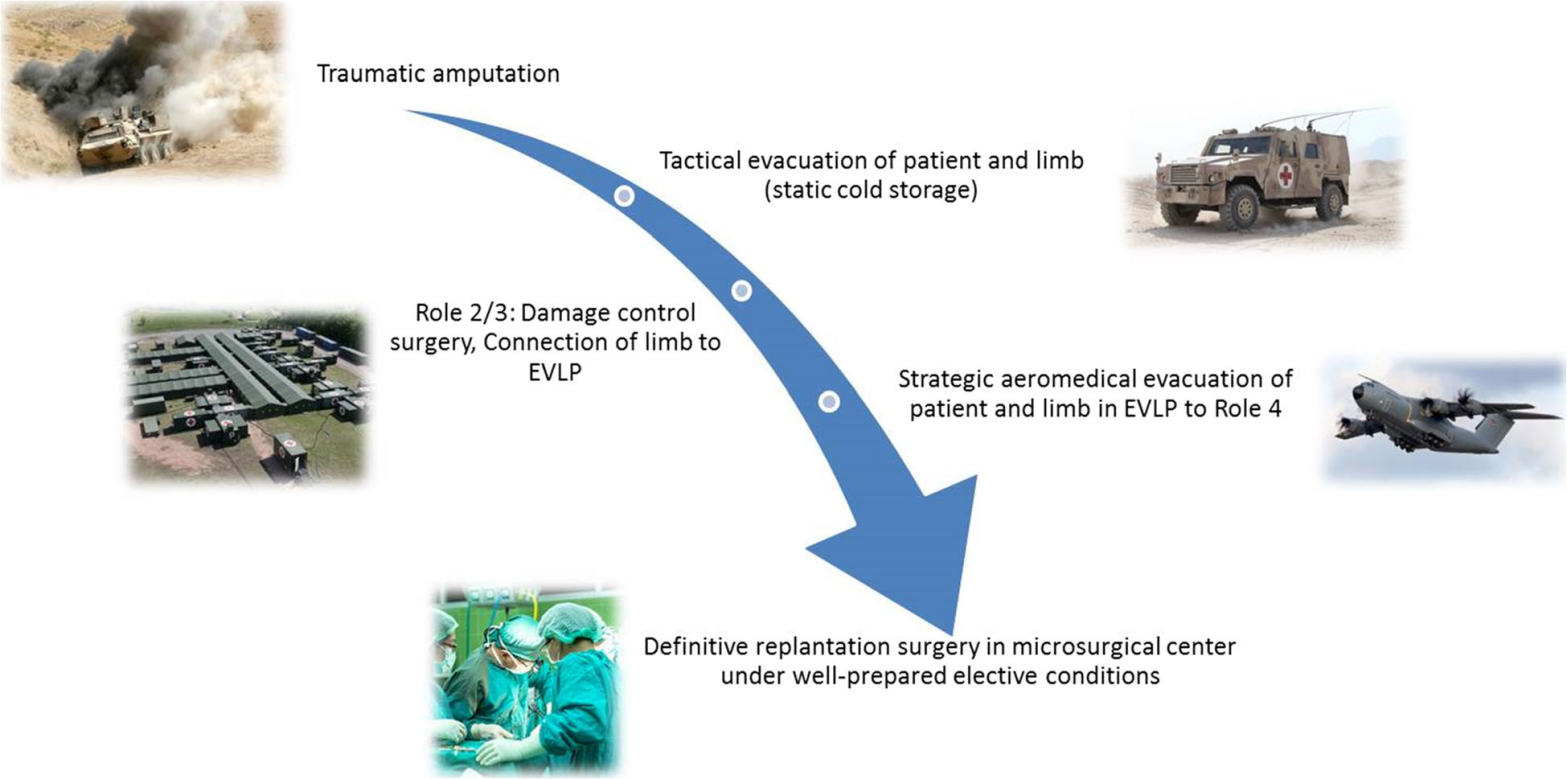
Hypothetical application in a fictive patient with combat-related limb loss and subsequent evacuation and treatment (EVLP: ex vivo limb perfusion)

Figure 2 shows the hypothetical application of EVLP in a fictive patient with combat-related limb loss and subsequent evacuation and treatment. After infliction of the injury, the patient is tactically evacuated to a Role 2 or Role 3 facility for damage control surgery. The amputated limb is preserved in static cold storage for tactical evacuation. The initial surgical treatment can completely focus on stabilizing the patient while the limb is connected to the ex vivo perfusion device and perfusion is started. Planning and execution of a strategic aeromedical evacuation to a Role 4 facility can be done without time pressure regarding limb ischemia time requirements. After admission to the Role 4 facility, a specialist microsurgical team can prepare the patient, as well as the limb, and then perform replantation surgery under highly elective conditions.

#### Proposed methodology

In terms of the actual in-field application of such a device, an algorithm would be as follows: After tourniquet treatment of the proximal stump of the severed limb, the amputated limb would be cleaned with disinfectants, rinsed with sodium chloride and sterilely placed on the perfusion device’s tray. Blunt dissection of the distal stump allows demonstration of the main vessels, which most military surgeons are familiar with for shunting procedures. A single use cannulation kit will be used to cannulate the arterial vessel and will be tied with either a suture or a snug clamp. The cannula will then be sterilely connected to the perfusion device, with free venous outflow. Hypothermic perfusion with off-the-shelf acellular perfusion solutions such as Perfadex or Steen will be initiated. For the trained person, the entire procedure will take approximately 15 min.

### Testing the hypothesis

First, the optimal conditions for long-term EVLP need to be found. Studies on the metabolic condition under perfusion in regard to cell damage and reactions of different additives to the perfusion solution should enlighten this aspect. The influence of different perfusion solutions on all affected tissues needs to be investigated, as well as the addition of medications (e.g., antibiotics or corticosteroids), nutrition, and electrolytes. Furthermore, the optimal oxygen saturation and temperature must be defined.

As mentioned above, there are data available from in vivo studies in porcine models, although the results are limited by the short perfusion time of 24 h. In the military setting, notably longer perfusion times need to be addressed. Therefore, future animal studies must focus especially on long-term perfusion, since this represents the military setting, considering the time for stabilization of the patient until evacuation to a tertiary treatment center. It is of utmost importance to allow the shift from a katabolic to an anabolic state of metabolism in the amputated limb.

**Fig. 3:**
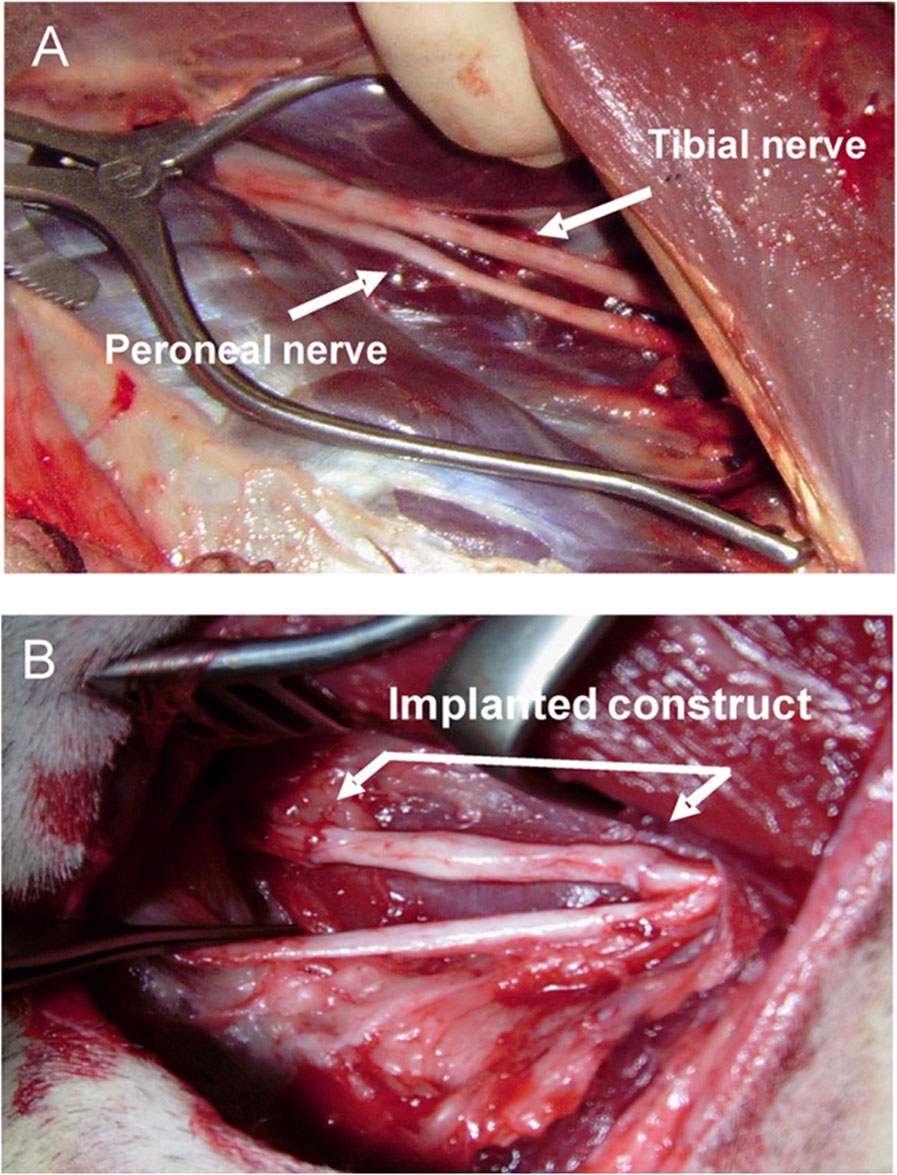
Demonstration of lesion side and nerve defect induction of peripheral nerve and construct implantation in adult sheep*. The tibial and peroneal nerves (arrow heads) were exposed (**a**). Nerve defect in tibial nerve of 6 cm and bridging of nerve defect lesion with vein/spider silk construct (**b**), which is sutured between the proximal and distal nerve stumps of the 6 cm nerve defect in adult sheep. *Figure by Radtke C, Allmeling C, Waldmann K-H, Reimers K, Thies K, Schenk HC, et al. Spider silk constructs enhance axonal regeneration and remyelination in long nerve defects in sheep. PLoS ONE 6(2): e16990. 10.1371/journal.pone.0016990 and used under the terms of the Creative Commons Attribution License

Restoring the functionality of the replanted limb should be a major aim for future research. In our opinion, the identification and improvement of all relevant factors for nerve regeneration are the key for successful aftercare. Nerves are especially prone to ischemia-induced damage which would occur within a timeframe of 2 h. Modern day VCA results impressively show that functionality can be excellent when the procedure is well prepared and performed under optimal conditions [13]. Systematic translational and multidisciplinary research projects should focus on axonal growth, nerve guidance structures and microsurgical nerve reconstruction. Promising data are available from two interesting developments in the field of nerve regeneration. The application of spider silk as a guiding structure for nerve regeneration is an upcoming innovation. Making use of spider silk is an idea dating back thousands of years [32]. For example, it is known that cobwebs were used to stop bleeding in ancient Rome, and natives of the Salomon Islands have been using spider silk-woven fishing lines for the last 300 years to catch small fish from their boats [33, 34]. There is strong evidence from a long-distance peripheral nerve gap model that spider silk enhances Schwann cell migration, axonal regrowth and remyelination, including electrophysiological recovery resulting in functional recovery (see Fig. 3) [35]. The growth of human neurons on spider silk has been established and there has actually been active neural cell migration and adhesion of neuronal cells to spider silk fibers [36]. Therefore, it needs to be investigated whether the early coupling of nerve ends to spider silk guiding structures during EVLP can lead to an increased rate of axonal growth after replantation and allotransplantation.

Additionally, EVLP provides the opportunity to apply another innovative approach. Current research is focused on the application of the biological polysaccharide chitosan and chitosan-coated nerve conduits. For short defects, chitosan-coated nerve conduits have already been approved for clinical applications, and preoperative nerve protection with such a conduit might lead to better results in the long run. Schwann cells grow exceptionally well on chitosan-coated surfaces, and co-cultivated neurons show increased neurite development [37]. There is further evidence that monocytes from peripheral blood have an increased expression of M2 phenotypes when in contact with chitosan [38]. Taken together, these factors might improve nerve regeneration and could lead to better functional outcomes.

The already available data should be complemented with analyses of longer periods of time. The profiles including the expression of hypoxia-induced genes, the cytokines and the anti-inflammatory and immunomodulatory factors at molecular level would help elucidate the effects further. One focus might be the evaluation of pharmacological approaches, such as the addition of PHD inhibitors [27]. The already available and promising results regarding MHC silencing should be researched in depth, especially regarding the major implications for its application in VCA.

A clinically applicable prototype needs to be developed in close cooperation with medical engineers and perfusion specialists. There has already been experience with solid organ machine perfusion and extracorporeal membrane oxygenation, which could be very useful during the prototype development phase. The EVLP device needs to address several specifications to allow its application in the military setting. It needs to be easy to use in stressful situations, unaffected by severe climate conditions and easy to transport, especially via air transport. Thus, the prototype must be intensively tested in the field to allow reliable clinical use. Such a device could also be a breakthrough for the field of VCA.

### Implications of the hypothesis

The development and clinical introduction of EVLP can address two challenges of modern medicine. First, there is a huge demand for VCA in civilian medicine. Such a device could be a breakthrough, by allowing a reasonable timeframe to organize a transplant procedure, including patient selection, graft allocation and technical and surgical preparation. Moreover, due to different trauma mechanisms in the civilian setting with less soft tissue damage than in combat injuries, the chance of successful replantation surgery is, per se, more likely. The Injury Severity Score, which is a practical tool for depicting the overall trauma burden, is significantly lower in civilian traumatic amputations [39]. First and foremost, we believe that this is the most relevant improvement for civilian medicine associated with EVLP. Second, EVLP might have a relevant impact on military medicine due to the outlined hypothetical implication in extremity salvage after traumatic amputation, although the rate of salvageable extremities is surely lower than that in civilian trauma due the different trauma mechanisms. Nevertheless, there is a notable number of wounded soldiers who could benefit from the application of EVLP, either in the acute setting of traumatic amputation or during delayed reconstruction with VCA. Injury patterns in the combat setting are associated with a high degree of soft tissue trauma and a significant loss of tissue. In these cases, VCA is a valuable treatment option, and the widespread availability of VCA programs could be possible on the basis of EVLP. Furthermore, recent evidence has shown that chronic pain and the failure of motor neuron regeneration lead to delayed amputations in patients with combat injuries [40]. Therefore, it can be hypothesized that the introduction of EVLP in the military setting could lead to a drastic reduction in the number of service members disabled by amputation. There is strong evidence that both civilians and military personnel will have improved outcomes regarding psychosocial factors and functionality after replantation surgery when compared to limb salvage or prosthetic treatment.

The availability of a trained microsurgical team that can carry out a sophisticated replantation procedure is unlikely in most kinds of military operations. EVLP enables replantation surgery in a Role 4 facility in the home country and changes the clinical setting from a highly urgent, life-threatening situation to a highly methodical, well-prepared starting point for optimal treatment of the wounded service member.

In conclusion, with the introduction of EVLP, the principle of “life before limb” will change to “life before limb before elective replantation or transplantation after EVLP”, from which both military and civilian societies will benefit.

## Data Availability

Not applicable.

## Abbreviations

EVLP: Ex vivo limb perfusion
PHD: Prolyl hydroxylase domain
IRI: Ischemia and reperfusion-related injuries
LEAP: Lower Extremity Assessment Project
METALS: Military Extremity Trauma Amputation/Limb Salvage
MHC: Major histocompatibility complex
VCA: Vascularized composite tissue allotransplantation
